# The DNA methylation landscape of hematological malignancies: an update

**DOI:** 10.1002/1878-0261.12744

**Published:** 2020-07-03

**Authors:** Pedro Blecua, Laura Martinez‐Verbo, Manel Esteller

**Affiliations:** ^1^ Cancer Epigenetics Group Josep Carreras Leukaemia Research Institute (IJC) Barcelona Spain; ^2^ Centro de Investigación Biomedica en Red Cancer (CIBERONC) Madrid Spain; ^3^ Institució Catalana de Recerca i Estudis Avançats (ICREA) Barcelona Spain; ^4^ Physiological Sciences Department School of Medicine and Health Sciences University of Barcelona Spain

**Keywords:** cancer, epigenetics, hematology, leukemia, lymphoma, methylation

## Abstract

The rapid advances in high‐throughput sequencing technologies have made it more evident that epigenetic modifications orchestrate a plethora of complex biological processes. During the last decade, we have gained significant knowledge about a wide range of epigenetic changes that crucially contribute to some of the most aggressive forms of leukemia, lymphoma, and myelodysplastic syndromes. DNA methylation is a key epigenetic player in the abnormal initiation, development, and progression of these malignancies, often acting in synergy with other epigenetic alterations. It also contributes to the acquisition of drug resistance. In this review, we summarize the role of DNA methylation in hematological malignancies described in the current literature. We discuss in detail the dual role of DNA methylation in normal and aberrant hematopoiesis, as well as the involvement of this type of epigenetic change in other aspects of the disease. Finally, we present a comprehensive overview of the main clinical implications, including a discussion of the therapeutic strategies that regulate or reverse aberrant DNA methylation patterns in hematological malignancies, including their combination with (chemo)immunotherapy.

## Graphical abstract

Epigenetic regulation of cell development and fate is a very complex and intricate process. Here, we address in detail the role of DNA methylation remodelers in hematological malignancies and their relationship with other epigenetic effectors. We include a critical discussion of available therapies and their clinical implications for these types of neoplasms, including the combination of hypomethylating and immunotherapy agents.

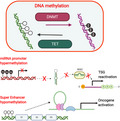

**Abbreviations**
2HG: 2‐hydroxyglutarate
5mC: 5‐methylcytosine
aKG: alpha‐ketoglutarate
AZA: azacitidine (5‐azacytidine)
DAC: decitabine (5‐aza‐20‐deoxycytidine azacytidine)
DNMT: DNA methyltransferases
FH: fumarate hydratase
GoF: Gain of function
HDAC: Histone deacetylases
HMA: hypomethylating agent
hmC: 5‐hydroxymethylcytosine
HSC: Hematopoietic stem cell
IDH: isocitrate dehydrogenase
lncRNA: long noncoding RNA
LoF: loss of function
miRNA: microRNA
SAH: *S*‐adenosylhomocysteine
SAM: *S*‐adenosylmethionine
SDH: Succinate dehydrogenase
TET: ten‐eleven translocation methylcytosine dioxygenase
TME: tumor microenvironment
TSG: tumor suppressor gene

## 1 Introduction

Epigenetic modifications are very common in our genomes. The most common consist of chemical additions to DNA bases (cytosines, within the CpG dinucleotide context) or histones (acetylation, methylation, phosphorylation) at the DNA level or post‐transcriptionally, respectively. Although these modifications are heritable and do not alter the chemical nature of their substrate, they may also have profound effects on their function, specifically by acting as ‘genetic switches’, regulating or turning on or off the expression of genes (in the case of DNA methylation), or closing or opening chromatin (in the case of histones). The latter phenomena may often provoke the complete rewiring of transcriptional programs. These two processes are commonly intertwined. For instance, acetylation of histones leads to a more open state of chromatin, a lower level of nucleosome occupancy and DNA hypomethylation patterns, favoring transcription [1]. However, epigenetic modifications are not restricted to DNA methylation or histone modifications. Therefore, epigenetics could be more precisely defined as the set of molecular phenomena that are not accompanied by genetic lesions that nevertheless have important effects on gene regulation and function and that are heritable. Long noncoding RNAs (lncRNAs) and microRNAs (miRNAs) exemplify this broader definition. The latter, a family of single‐stranded, ‘looped’ RNAs, tightly control and downregulate the expression of many genes under normal homeostatic conditions [2].

Technological advances (whole genome sequencing, WGS) and the advent of novel and improved sequencing technologies (assay for transposase‐accessible chromatin using sequencing; and Hi‐C) have prompted recent efforts to link genetic alterations in epigenetic effectors to cancer, specifically in hematological malignancies [3, 4]. These mutations include coding and noncoding areas of the DNA, as well as chromosomal rearrangements and the identification of novel chromatin structures. In this way, several authors have shown how these genetic lesions affect key epigenetic regulators in leukemia and other hematological neoplasms that lead to its appearance and progression. These include the following: alternative splicing events (e.g., U2AF1‐Ser34 and SF3B1‐K700E mutants) [4], aberrant methylation of superenhancers of known tumor suppressor genes (TSGs), such as *PAX5*and *GATA2*, and the activation of novel or known oncogene superenhancers (e.g., *TAL1*, *EVI1*, *MYC*), as originally reported by Heyn *et al*. [5], aberrant 3D chromatin topologies (e.g., disruption of the cohesin/CTCF complex); and dysfunctional lysine histone demethylases (e.g., mutant LSD1 and LSD2) [4].

Analogously, DNA methylation anomalies that arise during hematopoiesis may trigger the initiation and progression of hematological cancers. The pattern of DNA methylation at cytosine residues in the CpG sequences is established during early hematopoietic development and is heritable [6]. In humans, ~ 70% of CpG dinucleotides are methylated, even though the frequency of the CpG dinucleotide (3–8% of all cytosines) is relatively low in most of the human genome because of CG suppression (spontaneous C‐to‐T conversion), a process that is tightly controlled by the cell. When it fails in this purpose, the cell may undergo pathogenic transformations, through the epigenetic silencing of crucial TSGs or the unleashed expression of pro‐oncogenic genes (oncogenes), which therefore promote the onset and/or progression of cancers, including hemopoietic malignancies. However, DNA methylation aberrations do not occur as single events, but rather often appear in synergy with other epigenetic lesions (e.g., with histone modifications) [7]. This shows that the epigenetic regulation of cell development and fate is a very complex and intricate matter. Furthermore, DNA methylation abnormalities might have profound effects on the cytotoxicity of certain immune system cellular subtypes (e.g., by inducing the immunosuppressive, T‐exhausted phenotype). This has recently enabled researchers to design drugs that exploit the combined use of epigenetic therapies and novel immune checkpoint inhibitors (ICIs).

This work focuses on aberrant DNA methylation patterns in blood cancers, as there are already many studies of the connection between genetic lesions and epigenetic contributors; these may be found elsewhere [8, 9]. We discuss the main causes and clinical implications of malignant DNA methylation patterns, as well as their cellular regulators. This review is organized as follows: In Section 1, we summarize what is currently known about the connection between DNA methylation in normal and aberrant hematopoiesis; in Section 2, we discuss the intertwining of cellular regulation and DNA methylation, specifically the latter's interaction with the tumor microenvironment (TME) and the cell's metabolism (ME); in Section 3, we consider the link between DNA methylation and other epigenetic effectors, namely lncRNAs, miRNAs, and histone modifications; and finally, in Section 4, we comment in detail on the main clinical implications of all the above, with special emphasis on hypomethylating agents (HMAs) and combinations of drugs, including ICIs.

## 2 DNA methylation and hematopoiesis

### 2.1 DNA methylation in normal hematopoiesis

**Fig. 1:**
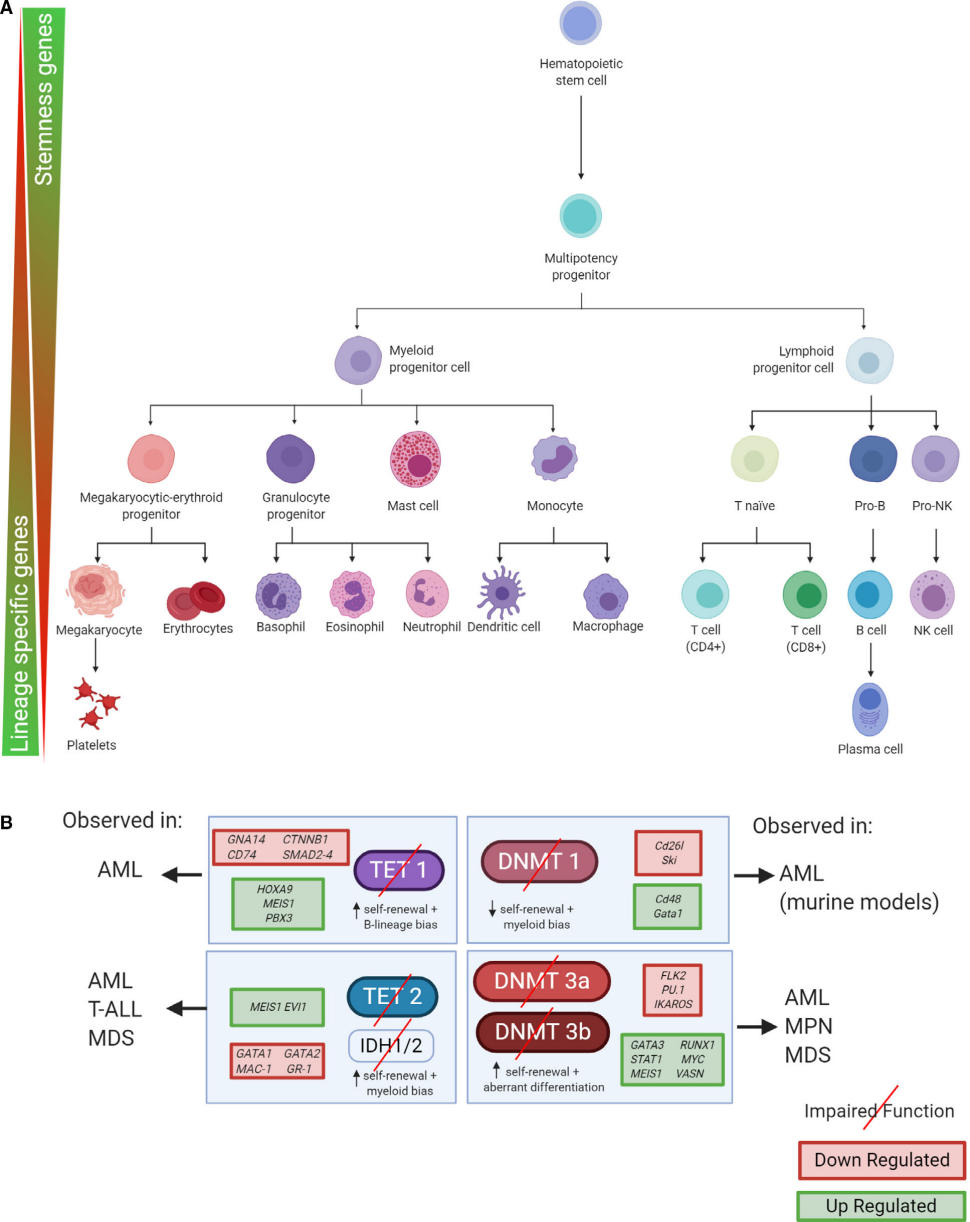
DNA methylation and hematopoietic development. (A) Schematic of the ‘classical’ view of hematopoiesis, where starting from a HSC, the whole blood cell population is formed in every subsequent step (binary bifurcation points). Epigenetics plays an important role in regulating both the myeloid and lymphoid lineages. Genes with key roles in HSC self‐renewal and pluripotency (termed stemness genes in the picture) are preferentially expressed at early stages of the process. On the contrary, as the different lineages are selected, the respective lineage‐specific genes are expressed accordingly. (B) In hematological malignancies, the epigenetic patterns present under homeostatic control become aberrant and the cells may suffer malignant transformations in every stage of the process. We illustrate schematically the main consequences of key effector methylation enzymes' malfunction and their impact in self‐renewal, lineage bias, and differentiation, along with some examples of the resulting up‐ or downregulated target genes.

Normal homeostatic control of hematopoiesis is a dynamic and tightly spatiotemporally regulated process by which the various types of terminally differentiated mature blood cells are formed [10, 11]. This takes place in the bone marrow, starting with hematopoietic stem cells (HSCs), a set of progenitor cells with the ability to self‐renew and differentiate into the various types of blood cells, each of which has different functionalities and biological properties. Even though the precise molecular mechanisms by which lineage commitment occurs are currently the subject of intense debate [12, 13, 14], it is well established that epigenetics is important for regulating HSCs during every step of its transformation, and influences self‐renewal, differentiation, and the developmental fates of the various hematopoietic progenitor cells (i.e., myeloid and lymphoid lineages, [15], see Fig. 1). Specifically, methylation analyses have shown how the epigenetic map changes within the different stages of HSC differentiation [16, 17]. DNA methylation marks are very stable and are passed on through daughter cells through differentiation, making it an effective tool for cell lineage reconstruction [18, 19]. The vast majority of methylation events actually occur in CpG‐poor regions, in normal and neoplastic tissue [20]. In the former, the majority of these sites are additionally located distal to transcription start sites (TSSs), like in (super)enhancers [5, 21]. It has been shown in murine models that DNA methylation patterns are very dynamic, with different and characteristic patterns for myeloid and lymphoid lineages [22]. However, according to a complementary hematopoiesis model suggesting that HSCs might actually have a continuum of differentiation stages [23], emerging evidence shows cell lineage conversion, often as a mechanism to acquire resistance to therapeutic agents. Along these lines, Bueno‐Costa *et al*. [24] very recently showed how DNA (hypo)methylation is crucial for pre‐B‐acute lymphoblastic leukemia (ALL) cell transdifferentiation into functional macrophages, affecting both cis‐ and trans‐acting gene regulatory elements of the genome.

During early stages of HSC development, genes associated with stemness (self‐renewal and multipotency) are open for transcription, whereas genes related to specific functions and lineages remain (epigenetically) silenced (see Fig. 1A). During successive differentiation stages, genes associated with specific lineages start to be expressed, while, conversely, the cells' epigenetic machinery silences genes associated with pluripotency (see Fig. 1A). In hematological malignancies, these DNA methylation patterns may become aberrant and the cells may acquire malignant phenotypes during this process. Aberrant methylation usually originates from a loss or gain of function (LoF and GoF, respectively) of the different proteins and enzymes involved in the methylation process [DNMTs, ten‐eleven translocation methylcytosine dioxygenase (TETs), isocitrate dehydrogenase 1/2 (IDH1/2)], often due to their acquisition of clonal somatic mutations (including co‐occurrence with secondary mutations in other ‘synergy‐acting’ genes) or external factors affecting enzyme function (e.g., substrate limitation; see Fig. 1B). Lastly, as has been pointed out in recent years, the process of clonal hematopoiesis (including recurring mutations in DNMT3A, TET2, or IDH1/2) in otherwise healthy individuals might also predispose them to certain types of hematological cancers [25]. The main scope of this review is to provide an overview of these DNA methylation‐dysregulated processes, bearing in mind that they often act synergistically with other epigenetic aberrations, such as histone marks [26], and the deregulation of both lncRNA [27] and miRNA expressions [28].

### 2.2 DNA methylation in hematological malignancies

Epigenetic aberrations are common in hematological malignancies, and the latter are largely driven by extensive epigenome remodeling. LoF or misfunction in key DNA methylation‐related enzymes is widely observed in many types of hematopoietic neoplasms, such as myelodysplastic syndrome (MDS), myeloproliferative neoplasms (MPNs), acute myeloid leukemia (AML), T‐ALL and diffuse large B‐cell lymphoma (DLBCL; see Table 1). LoF mechanisms comprise mutation events and hypermethylation‐driven gene silencing. The resulting aberrant DNA methylation patterns are often lineage‐specific and are commonly accompanied by secondary mutations [29, 30]. Furthermore, coupled LoF of two such enzyme‐expressing genes can act synergistically (e.g., TET2 loss simultaneously occurring with DNAMT3A loss in AML) [31]. Interestingly, TET loss (increased hypermethylation) or DNMT loss (increased hypomethylation) can both lead to hypermethylation and hypomethylation of the promoters of target genes, upon careful examination of individual differentially methylated regions [32]. Nevertheless, epigenetic abnormal plasticity in hematological cancers does not exclusively arise from defects in the aforementioned epigenetic remodelers, and several other factors (or a combination thereof) might contribute to pathogenesis post‐transcriptionally. For instance, it has been recently shown how hypermethylation‐mediated silencing of the decapping enzyme NUDT16 [e.g., a protein that removes the N7‐methyl guanosine (m7G) cap at the 5′ end of gene transcripts] mediates c‐MYC activation in T‐ALL, both *in vitro* and *in vivo* [33]. Bearing this fact in mind, the main DNA methylation remodelers and the resulting impact of their impairment in blood cancers are considered below.

**Table 1:** DNMT, TET, and IDH observed mutations in hematological malignancies and their prognostic value.

Gene	Mutation	Condition	Frequency	Prognostic value	References
DNMT1	Missense and nonsense mutations	AML	Small subset of cases (rare mutations)	Not studied	[149, 150, 151]
DNMT3A	Missense mutation (amino acid R882H)	AML	20–60% (hot spot)	Adverse prognostic impact	[9, 150, 152, 153, 154]
		MDS	10%	Adverse prognostic impact	[155, 156]
	Frameshift and truncating mutations	AML	15–20%	Not studied	[9]
DNMT3B	Truncating mutations	AML	Small subset of cases (rare mutations)	Not studied	[157]
	Missense mutation (amino acid N442K)	ATL	Small subset of cases (rare mutations)	Not studied	[158]
TET1	Missense mutations	AML	~ 1%	Not studied	[40, 150]
	Missense and frameshift mutations	T‐ALL	14%	Not studied	[40, 159]
TET2	Several missense, nonsense, and frameshift mutations	AML	~ 10%	Shorter overall survival (mutated vs no mutated)	[41]
	Truncating mutations	MDS	10–30%	Not studied	[42, 43]
	Several missense, nonsense, and frameshift mutations	MPN	10–20%	Not studied	[41, 42, 43]
	Several missense, nonsense, and frameshift mutations	CMML	40–50%	Not studied	[41]
	Several missense, nonsense, and frameshift mutations	DLBCL	5–10%	Not observed	[117]
IDH1	Missense mutation (amino acid R132H)	AML	~ 10%	Controversial	[48, 58]
		MDS	2–10%	Controversial	[58, 160]
IDH2	Missense mutation (amino acid R172K)	AML	~ 10%	Controversial	[48, 58]
		MDS	2–5%	Controversial	[58, 160]
	Missense mutation (amino acid R140Q)	AML	~ 10%	Controversial	[48, 58]
		MDS	2–10%	Controversial	[58, 160]

#### 2.2.1 DNA methyltransferases

*DNMT3A, DNMT3B, DNMT3L* and *DNMT1* are collectively known as ‘epigenetic landscaping’ genes. The first two are *de novo* methyltransferases, depositing methylation marks over an otherwise unmodified DNA template. In turn, *DNMT1* is a maintenance methyltransferase, which acts by propagating already existing methyl marks upon DNA replication [9]. *DNMT3L* has no catalytic domain and is thought to couple with *DNMT3A*. All three enzymes play a pivotal role in mammalian development and hematopoiesis [9]. In murine models, the activity of the catalytic domain of WT *Dnmt1* is essential for HSC self‐renewal. Its impairment leads to downregulation of the self‐renewal‐associated genes *Cd26l* and *Ski* in murine models (see Fig. 1B). Likewise, when misfunction of *Dnmt1* occurs in these models, there is a clear bias of HSC differentiation toward the myeloid lineage, with a consequent reactivation of the myeloerythroid‐specific genes *Cd48* and *Gata1* [34]. In humans, very few mutations have been detected in this gene (AML) and occur at very low frequencies (see Table 1). Unlike its *DNMT1* counterpart, complete loss of *Dnmt3a* in mouse models has been shown to favor self‐renewal, through the DNA methylation‐induced dysregulation of multipotency‐related genes, such as *Runx1* and *Gata3* (see Fig. 1B). Defective HSC differentiation has also been observed in the same *Dnmt3a* null mice model, through hypermethylation of genes such as *Flk2* and *Ikaros* [32]. Mutations in this enzyme have been reported in AML and MDS patients. 20–60% and 10% percent of patients, respectively, harbor the hot spot mutation R882H (see Table 1) and are associated with an adverse prognosis. Furthermore, mutated DNMT3A patients were shown to undergo promoter hypomethylation and the subsequent upregulation of the leukemogenic HOX co‐factor MEIS1 [35]. Analogously, *Dnmt3b* knock‐out mouse models have a similar, but milder, phenotype than their Dnmt3a null counterpart. Mutations in DNMT3A have been reported in AML and MDS patients, with a prevalence of 20–60% (hot spot mutation R882H) and 10% percent of patients, respectively, and are associated with an adverse prognosis (see Table 1). A stronger phenotype is nevertheless achieved when *Dnmt3a* and *Dnmt3b* both lose their function. In both cases, DNMT3A/B deficiency results in a global hypomethylation pattern.

#### 2.2.2 Ten‐eleven translocation methylcytosine dioxygenases

Ten‐eleven translocation methylcytosine dioxygenase family proteins are DNA methylation regulators also involved in hematopoietic differentiation. It consists of three enzymes, *TET1*, *TET2* and *TET3*. They are able to convert 5‐methylcytosine (5mC) to 5‐hydroxymethylcytosine (5hmC) through oxidation, 5hmC to 5‐formylcytosine (5fC), and finally to 5‐carboxylcytosine (5caC), which eventually leads to loss of the 5mC mark and DNA demethylation [36, 37, 38]. They also depend on cellular alpha‐ketoglutarate (aKG) levels to function properly. *TET1* plays an indispensable role in hematopoiesis by regulating target genes that mediate leukemic transformation [39] and is considered to be a tumor suppressor (mutated in 14% of T‐ALL patients,see Table 1) [40] except in MLL‐rearranged leukemias, where it acts as an oncogene, facilitating leukemogenesis by being directly activated by MLL fusion proteins and upregulating the expression of key oncogenic target genes such as *HOXA9*, *MEIS1,* and *PBX3* [39]. Impairment through epigenetic silencing or missense and frameshift mutations (see Table 1) of the *TET1* enzyme has also been observed in myeloid malignancies, such as MDS, MPN, or AML [41, 42, 43], and is associated with lymphoid (B lineage) bias and increased HSC self‐renewal (see Fig. 1B). Genes downregulated by *TET1* loss include *GNA14, SMAD2–4,* and *CTNNB1* [40]. These genes are important in HSC homeostasis and transformation. Unlike *TET1*, which is often inactivated by epigenetic silencing, *TET2* LoF often occurs by mutational processes. Mutated *TET2* may contribute to the initiation of both myeloid and lymphoid malignancies [44]. In AML, 10% of patients have been observed to harbor mutations (missense, frameshift, and nonsense). By comparison, patients who are wild‐type (WT) for *TET2* had a better OS (see Table 1). However, we have a poor understanding of how epigenetic changes induced by its loss contribute to leukemogenesis. *TET2* mutations lead to enhancer hypermethylation (up to 25% of active enhancer elements) and to a critical deregulation of enhancer‐associated gene expression patterns in hematopoiesis [45]. *TET2* deletion is related to enhanced self‐renewal capacity and myeloid bias, as evidenced in a murine model‐based study that showed greater expression of the self‐renewal regulators *Meis1* and *Evi1*, and a lower level of expression of myeloid‐specific factors *Cebpa, Mpo,* and *Csf1* [46, 47].

#### 2.2.3 Isocitrate dehydrogenase 1 and 2

*IDH1* and *IDH2* are NADP+‐dependent enzymes that catalyze the interconversion of isocitrate and aKG. *IDH1* and *IDH2* often acquire neomorphic (GoF) mutations, which are thought to occur early in leukemogenesis [48]. This bestows novel catalytic activity on these enzymes, enabling them to convert aKG to its structural analog 2‐hydroxyglutarate (2HG) [49, 50]. In turn, this activity impairs the function of enzymes that require aKG as a substrate, like *TET2* (described above), inhibiting the hydroxylation reaction of 5mC by *TET2* [48]. Missense mutations in IDH1 (amino acid change R132H) or IDH2 (amino acid change R172K) have been observed in AML and MDS patient cohorts (see Table 1), but their clinical impact remains controversial. *TET2* LoF mutations and IDH GoF mutations are mutually exclusive in AML [48], suggesting that lesions in these genes may in fact be biologically redundant. *IDH1/2* GoF arises in AML through the hypermethylation of several transcription factors that control myeloid differentiation, such as *GATA1*, *GATA2,* and *EVI1* [48] and are associated with increased HSC self‐renewal (see Fig. 1B).

### 2.3 Intertwining of cellular regulation and DNA methylation

**Fig. 2:**
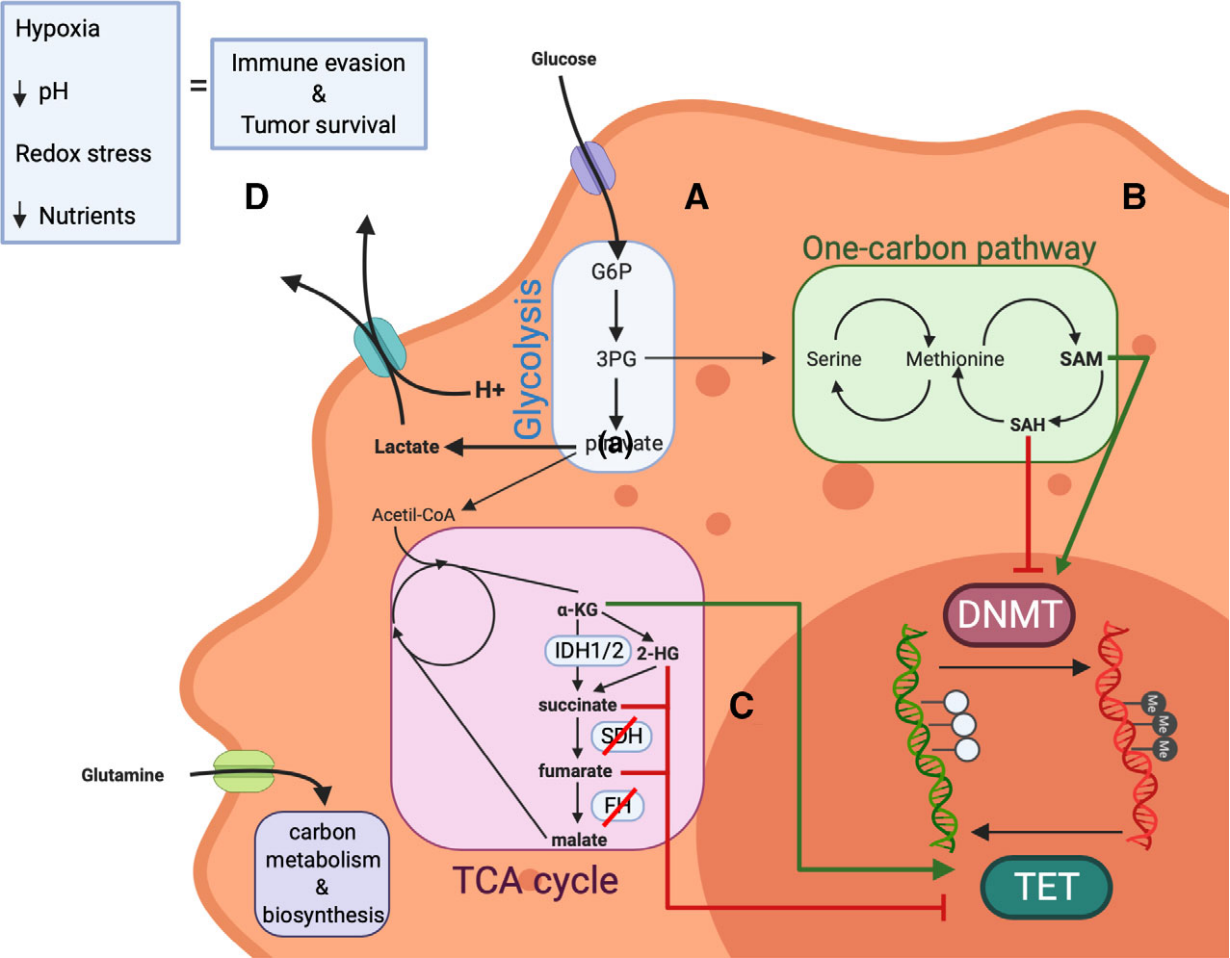
Cellular regulation of DNA methylation. DNA methylation influences neoplastic cell's metabolism and *vice versa*. (A) Glycolysis is regulated by, among others, the HIF1 pathway. A crucial TSG of this pathway, VHL, has been shown to be epigenetically silenced in hematological malignancies. See text for more details. (B) SAM is the substrate needed by DNMTs in order to methylate the DNA, and it is one of the limiting aspects that favors DNMT impairment in tumors. SAM is then converted to SAH, which usually accumulates and acts as an inhibitor of the process in the normal product‐negative regulation of the enzyme function. See text for more details. (C) Although the tumor cell prefers to transform glucose into lactate to rapidly obtain ATP, intermediate metabolites and redox power, TCA cycle intermediates play an important role in methylation as several of them act upon TET demethylases. TET enzymes use α‐KG as a substrate to actively demethylate DNA, and, as SAM, it is rather limited in the tumor cell. α‐KG can be transformed into 2‐HG by mutated forms of IDH1 or IDH2, which acts as a competitor of α‐KG and impairs TET function. SDH and FH might be silenced in hematological malignancies, which originates an accumulation of succinate and fumarate, which together with 2‐HG act as TET inhibitors in the cell. (D) Due to the increase in nutrient uptake, hypoxic conditions, redox stress, and environment acidification, the tumor cell creates an environment, which enhances tumor survival while it dampens immune cell activation, that is, favoring macrophage M2 polarization or Treg phenotype. 3PG, 3‐phosphoglycerate; G6P, glucose‐6‐phosphate; α‐KG, α‐ketoglutarate.

The cellular metabolism and the TME are both tightly regulated by aberrant cells in order to survive under different hostile environments, and they often influence each other. Neoplastic cells are known to increase nutrient uptake from their surroundings in order to maintain their high biosynthesis rate and division capacity. As these cells prefer to perform aerobic glycolysis, intermediate metabolites accumulate during this process, giving rise to by‐products such as 3‐phosphoglycerate, which aids the function of the cancer cell's one‐carbon pathway (see Fig. 2B). Also, cancer cells generate large quantities of lactate, which is usually released into the environment in conjunction with H^+^, with the consequent acidification of the environment (lower pH; see Fig. 2D). Below, we describe the known connections between metabolism, the TME, and DNA methylation in hematological malignancies.

### 2.4 DNA methylation and metabolism

The alteration of metabolic pathways in hematological cancers and other malignancies is an essential step in the aberrant growth and proliferation of neoplastic cells, which are supported by deregulation of the epigenetic machinery [51]. Moreover, an aberrantly reprogrammed metabolism also promotes changes in the epigenetic landscape. DNA methylation is involved in regulating three of the main metabolic pathways of cancer cells: glycolysis [52], one‐carbon and methionine pathway [53], and the tricarboxylic acid (TCA) cycle [54] (see Fig. 2A–C). DNA methylation is indirectly involved in preferential use of the aerobic glycolysis even in normoxia (the Warburg effect). This is supported by the fact that several tumor suppressors involved in pathways that control glycolysis activation are often epigenetically silenced by promoter hypermethylation, like the TSG *VHL* [55]. *VHL*, along with other TSGs, is involved in the HIF1 pathway, which is one of the pathways controlling the tumor's glycolytic activity [51] (see Fig. 2A). Hypermethylation of *VHL* in multiple myeloma (MM) patients [55] leads to transcriptional silencing of the gene and therefore decreased HIF‐1alpha proteolysis, suggesting a possible mechanism for increasing angiogenesis and altering the bone marrow microenvironment. In the case of the one‐carbon pathway (Fig. 2B), it was recently reported that perturbing methionine/*S*‐adenosylmethionine (Met/SAM) metabolism in mixed‐lineage leukemia‐AF4 cell lines caused the loss of expression and activity of the H3K79 methyltransferase DOT1L. In turn, DOT1L loss reduced overall cellular methylation potential (low SAM/SAH ratio; SAH, *S*‐adenosylhomocysteine; see Fig. 2B) and increased the apoptotic rate in those cell lines [56]. The authors confirmed their findings by pharmacologically inhibiting Met/SAM metabolism in a clinically relevant patient‐derived MLL‐R leukemia xenograft model, which resulted in increased survival. They attributed this result, in part, to the repression of the expression levels of DOT1L‐regulated leukemia‐inducing genes, such as *MEIS1* [56]. On the other hand, another study showed that inhibition of DOT1L induces apoptosis of DNMT3A‐mutated AML cells *in vitro* [57], resulting in diminished expression of key proleukemic genes, including *MEIS1*. Given all these findings, it is reasonable to speculate that alteration of the Met/SAM pathway and DOT1L inhibition may act synergistically to stop leukemogenesis, particularly in DNMT3A‐mutated and MLL‐rearranged AML. Furthermore, distortion of the TCA pathway is directly involved in hematological cancers (see Fig. 2C). This occurs in several types of myeloid malignancies, like MDS [58], where IDH1/2 mutations induce the conversion of aKG into the oncometabolite 2‐HG and therefore, as mentioned earlier, the impairment of proper aKG‐dependent DNA demethylase TET protein function. As a consequence, aberrant hypermethylation patterns are often observed in IDH1/2‐deficient hematological neoplasms [58]. Finally, inhibition of succinate dehydrogenase (SDH) and fumarate hydratase (FH) function in the TCA cycle may lead to aberrant methylation patterns, leading to decreased aKG levels, as in the case mentioned above, and TET misfunction (Fig. 2C) [59]. However, the latter phenomenon is, to our knowledge, poorly studied in hematological malignancies and more research in that direction is needed to demonstrate LoF patterns, mainly through hypermethylation, of SDH and FH.

### 2.5 TME and DNA methylation

DNA methylation changes, including hematological neoplasms, occur frequently in cancer. These changes help shape the TME, which in turn elicits immune tolerance and drug resistance. The interaction between DNA methylation and TME regulation is complex and has very recently attracted the attention of many groups [60]. In brief, neoplastic cells create an environment that enhances tumor survival while dampening immune cell activation. This is achieved by the tumor's increase in nutrient uptake and induction of low oxygen (hypoxia) and high lactate (environment acidification) levels in the TME (see Fig. 2D). Under these conditions, macrophages with the M1 immunoactive phenotype are often driven toward the M2 immunosuppressive phenotype [61, 62, 63, 64]. Analogously, the TME may inhibit cytotoxic T‐cell expansion and induce the consequent loss of their anticancer response [65]. The role of DNA methyltransferases (DNMTs) and demethylases (TETs) [66] in reshaping the immune system and immune suppression of the TME is being increasingly recognized. As an illustration of this, TET2‐deficient CD8^+ ^tumor‐infiltrating lymphocytes (TILs) were shown to display greater antitumor efficiency [67]. However, more research effort is needed in the coming years to fully elucidate the precise molecular mechanisms involved in this process.

### 2.6 DNA methylation and regulators of other epigenetic events

As mentioned earlier, DNA methylation aberrations are often accompanied by other epigenetic lesions in hematological cancers. As an illustration of these intricate and complex processes, in the next three sections we describe the link between DNA methylation and other epigenetic regulators. We focus on DNA methylation effects coupled with lnRNA activity, miRNAs deregulation and histone modifications, respectively, with a brief mention to (super)enhancers.

### 2.7 Long noncoding RNAs

**Fig. 3:**
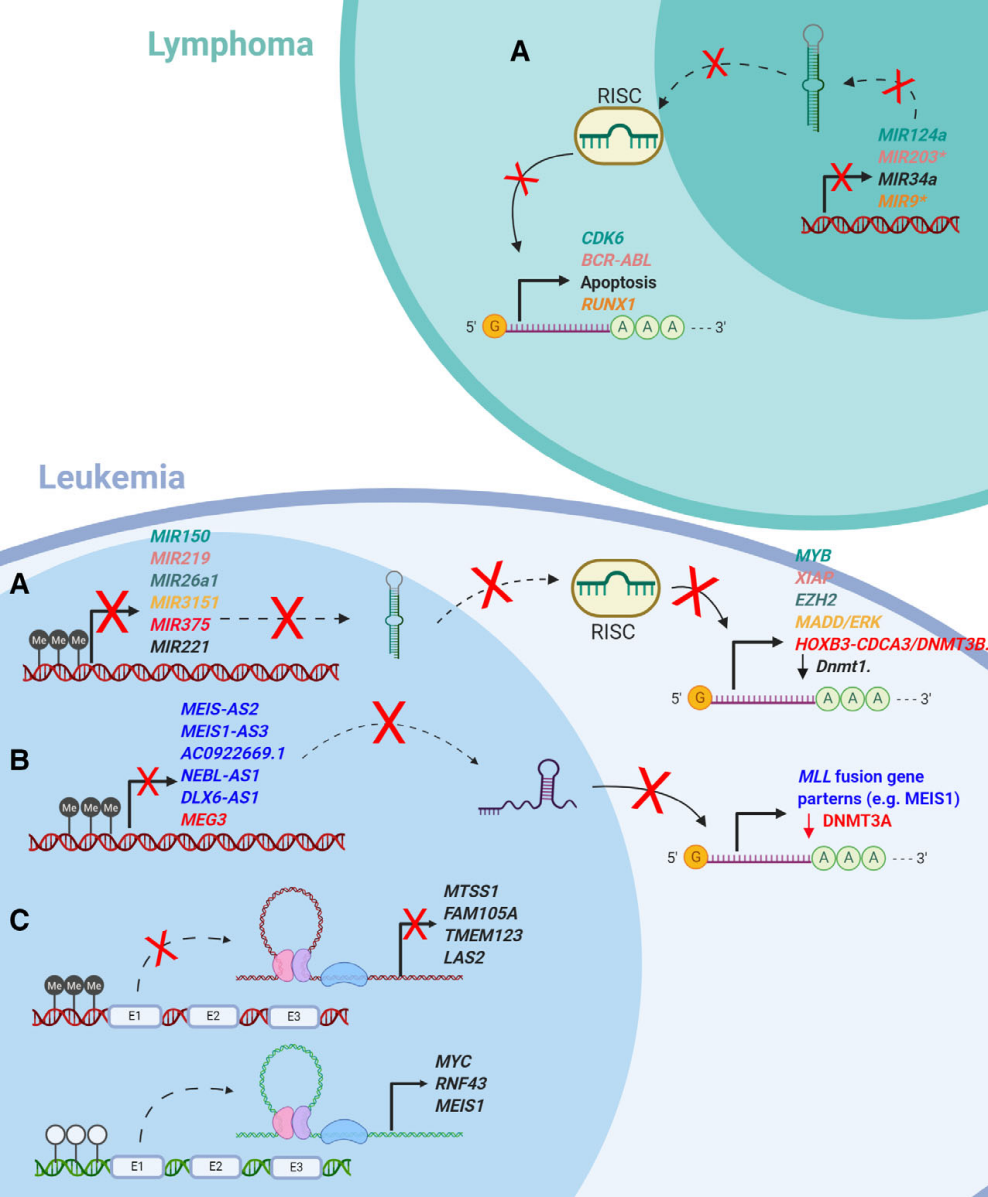
Impaired regulatory elements in leukemia and lymphoma. Different miRNA/lncRNA colors (left) match the corresponding up/downregulated genes (right). Methylation of miRNA/lncRNAs promoters in the figure has all negative impact in the disease, except for the lncRNA MEG3, which has been shown to suppress leukemogenesis (see text). miRNAs might be both TSG and oncogenes, as specified in the text. miRNA genes marked with (*) are also found hypermethylated both in lymphoma and in leukemia (A) miRNAs genes are small noncoding RNA fragments that usually interact with the target mRNA and repress its translation. In leukemia and lymphoma, several miRNAs' promoter regions have been described to be hypermethylated, which allows the target genes to be expressed. (B) lncRNA are long RNA fragments that by interacting with their target can interfere in several stages of target expression and function. In the figure, we show how hypermethylation of the promoter region of different genes allows that several members of *MLL* fusion gene family to be translated contributing to leukemia progression. (C) Superenhancers are DNA elements, which by loop formation allow an increase in the production of its targets. In leukemia, enhancers of TSGs are hypermethylated, whereas oncogene's enhancers are found to be hypomethylated. E, enhancer; Me, 5‐methyl cytosine; RISC, RNA‐induced silencing complex.

Long noncoding RNAs and small noncoding RNAs (e.g., miRNAs) play a key role in the development and progression of leukemia [68], and are therefore new biomarkers and potential targets for novel therapies [69]. lncRNAs are nonprotein coding RNAs longer than 200 bps. They critically regulate gene expression and are highly tissue‐specific [70]. Alterations in the expression of lncRNAs are thought to affect the onset and development of various hematological malignancies by modulating critical cellular pathways of HSC development [4, 71]. While several studies have recently highlighted the involvement of lncRNAs in blood neoplasms [72], to our knowledge, little is known about the regulation of lncRNAs through DNA methylation in these type of cancers, which is the main focus of this review. One such example is the tumor suppressor lncRNA MEG3 hypermethylation, reported in AML [73]. MEG3 downregulates DNMT3A via MDM2/RB signaling to suppress leukemogenesis [74] (see Fig. 3B). Another study [75] identified the role of (gene body) hypermethylated anti‐sense lncRNAs (AS‐lncRNAs) in ALL, including the lncRNAs MEIS1‐AS2, MEIS1‐AS3, AC092669.1, NEBL‐AS1, and DLX6‐AS1. The resulting repressed genes are MLL fusion genes, such as *MEIS1* (See Fig. 3B). The authors proposed a putative oncogenic role of these genes independent of the MLL fusion partner. James *et al*. [76] conducted a systemic study of the influence of lncRNAs in B‐cell ALL patients at initial diagnosis and at relapse. They focused on the main disease subtypes and characterized the link between diagnosis‐ and relapse‐specific lncRNAs based on the differential expression and differential methylation patterns between the two conditions and within subtypes. They identified previously known (relapse‐specific) onco‐lncRNAs that were promoter‐hypomethylated in at least one of the subgroups, including RP11‐701P16.5 and SLC38A3, as well as TCL6 and LINC00312, which were observed to be promoter‐hypermethylated. Furthermore, they found lncRNAs with a novel role in B‐cell ALL, namely R11‐138M12.1 and RP11‐624M8.1, that were significantly hypomethylated at their promoter region and transcriptionally upregulated in one of the subgroups. We summarize some of these and other examples of novel (de)methylated lncRNAs in Fig. 3B, as well as novel DNA methylation‐induced deregulation of (super)enhancers (Fig. 3C), such as those corresponding to MYC and RNF43, whose role in hematological malignancies is being increasingly recognized [5].

### 2.8 microRNAs

microRNAs are a set of regulatory ncRNAs, about 22 nucleotides long, that usually repress the transcription of their target genes [77]. They are crucial to physiological and pathological processes such as cell differentiation and inflammation, and to the pathogenesis of major hematological malignancies, such as AML [78]. miRNAs may regulate DNMT activity, and on the other hand, hypermethylation of miRNA promoters may inhibit their function, which can be either tumor‐suppressing or oncogenic [72]. An example of miRNA deregulation caused by aberrant methylation is miR‐29b tumor suppressor upregulation in bone marrow cells, which reduces the expression of DNMTs, enhancing the activity of demethylating agents (decitabine) and improving the remission rate of AML patients, reactivating the other TSGs [79]. On the other hand, inhibition of the onco‐miRNA‐221 could directly reactivate TSGs and, at the same time, upregulate the expression of other TSGs by downregulating Dnmt1 activity in mice [80] (see Fig. 3A). As mentioned above, the promoters of certain miRNAs may also be targeted by DNMTs, causing their hypermethylation (in the case of tumor suppressor activity). Along these lines, HOXB3 enhanced DNMT3B binding to the promoter of the tumor suppressor miR‐375, leading to DNA hypermethylation and a lower level of expression of miR‐375 [81] (see Fig. 3A), thereby predicting poorer outcome in AML. In Fig. 3A,C, we summarize recent findings of these and other pre‐miRNA promoter regions in hematological cancers, which are found to be hypermethylated, as well as their molecular impact on the respective diseases, where these have been observed.

### 2.9 Association between histone modifications and DNA methylation patterns

It is not currently fully understood how histone modifications affect DNA methylation and *vice versa*. Aside from aberrant DNA promoter hypermethylation or hypomethylation, histone modifications are recognized as important mechanisms in cancer initiation and progression, including those of hematological cancers [4, 82]. These modifications are of three main types—acetylation, methylation, and phosphorylation—all of which occur post‐translationally. As with DNA methylation patterns, they may activate or repress transcription. For instance, histone‐3/4 acetylation leads to a more open chromatin state, favoring transcription [83]. However, histone 3 methylation may lead to transcriptional repression or activation [84]. Links between DNA methylation patterns and histone modifications in hematological neoplasms (and cancer in general) have been reported. In hematological cancers, histone marks accumulated on promoters are often enriched in H3k27me3 alone or in combination with H3K4me3 (bivalent promoters) [85]. Both of these histone modifications are negatively correlated with DNA methylation and transcription regulation. H3K27me3 is associated with promoter hypermethylation and gene silencing [86] in HSCs, while H3K4me3 is usually associated with hypomethylation of promoters and gene upregulation [87]. However, recent studies suggest that H3K4m3 and DNA hypermethylation also have a synergistic function [88]. For the sake of clarity, we specifically focus here on H3k27me3, whose proper maintenance is known to be critical for the homeostasis of normal cells, and whose deregulation often leads to aberrant hematopoiesis [4]. Trimethylation of histone H3K27 (H3K27me3) is mechanistically linked to the polycomb group (PcG) proteins through its core member, EZH2, which has histone methyltransferase activity with substrate specificity for H3K27 through its SET domain (as do other core components of the PRC2, acting with EZH2, such as EED and SUZ12) [4]. The histone methyl mark imposed by PRC2 is strongly associated with transcriptional repression [89]. Consistent with these observations, PRC2 has been shown to be associated with *de novo* DNMT activity and, consistent with this, H3K27me3 and PRC2 targets are known to be positively correlated with DNA (hyper)methylation [90, 91]. However, PRC2 may function as a tumor suppressor or an oncogene in leukemogenesis and lymphomagenesis, depending on the biological context. LoF mutations in EZH2 are associated with poor survival in MDS and MPNs [92], highlighting the role of the PRC2 complex as a tumor suppressor. On the other hand, GoF mutations or overexpression of EZH2 may often lead to a more aggressive disease phenotype, repressing tumor suppressor CDKN2A expression, which in turn is linked to HSC proliferation and self‐renewal [93], like in the case of MLL‐rearranged leukemias [94], suggesting that the PRC2 complex may act as an oncogene. With respect to the latter, that is, GoF EZH2 mutations, relatively recent research provides evidence of that the clinical utility and development of small molecule EZH2 inhibitors provide a means to arrest aberrant leukemic/lymphoid transformation in certain hematological malignancies. Future research effort is needed to evaluate the usefulness of the combined action of EZH2 and DNMT inhibitors in blood cancers, as recently reported in MM therapy‐resistant cell lines [95].

### 2.10 Clinical implications

As described above, it is increasingly evident that epigenetic modifications play a major role in aberrant hematopoiesis. DNA methylation is one of the main processes whose deregulation leads to malignant cell transformation and progression in hematological cancers. These aberrant modifications in DNA (de)methylation‐inducing genes may be reversed and are thus attractive clinical targets. Furthermore, disease‐specific DNA methylation signatures have been developed within the last decade in an attempt to stratify patients and therefore personalize their treatment to achieve improved outcomes. In this last section, we will briefly describe the main drugs (either FDA‐approved or being clinically trialed) that reverse these aberrant methylation patterns, either alone or in combination with other targeted therapies and including immunotherapy (Table 2), as well as their clinical relevance and implications. We will also review the prognostic methylation signatures developed to date with respect to the various hematological neoplasms.

**Table 2:** (Pre)Clinical status of current DNA methylation‐related drugs in combination with other epigenetic therapies or chemoimmunotherapy. BP, blast phase; CR: complete remission; DHFR, dihydrofolate reductase; G‐CSF: granulocyte colony‐stimulating factor; RR, refractory/relapse; TK, tyrosine kinase.

Drug	Target	Target domains	Condition	Current clinical status	NTC number	Reference
AZA + belinostat	DNMT/HDAC	DNA methylation/histone deacetylation	AML	Completed	NCT00351975	–
AZA + entinostat	DNMT/HDAC	DNA methylation/histone deacetylation	AMLMDS	Phase II, completed	NCT00313586	[161]
AZA + entinostat	DNMT/HDAC	DNA methylation/histone deacetylation	AML (old)	Phase II, recruiting	NCT01305499	–
AZA + homoharringtonine	DNMT/TET	DNA methylation/DNA demethylation	AML	Phase III, recruiting	NCT04248595	–
AZA + pinometostat	DNMT/DOTL1	DNA methylation/histone methylation	AML	recruiting	NCT03701295	–
AZA + pracinostat	DNMT/HDAC	DNA methylation/histone methylation	AML	Phase II, completed	NCT01912274	[162]
AZA + vorinostat + gemtuzumab ozogamicin	DNMT/HDAC/CD33	DNA methylation/histone methylation/Leukemic cell marker	AML	Phase I/Phase II, completed	NCT00895934	[163]
ASTX727 + FT‐2102	DNMT/IDH‐1	DNA methylation/cell metabolism	RR‐MDS RR‐AML	Phase I/Phase II, recruiting	NCT04013880	–
Decitabine + LBH589	DNMT/HDAC	DNA methylation/histone deacetylation	AML MDS	Phase I/Phase II, completed	NCT00691938	[164]
Decitabine + vorinostat	DNMT/HDAC	DNA methylation/histone methylation	RR‐AML AML MDS	Phase I, completed	NCT00479232	[165]
Decitabine + vorinostat	DNMT/HDAC	DNA methylation/histone deacetylation	RR‐AML	Phase II, terminated	NCT00882206	[166]
Decitabine + vorinostat + filgrastim + fludarabine + cytarabine	DNMT/HDAC/G‐CSF/DNA and RNA polymerases	DNA methylation/histone deacetylation/Neutrophil growth/DNA synthesis	RR‐AML	Phase I, recruiting	NCT03263936	–
Decitabine + vorinostat + filgrastim + fludarabine + cytarabine + sorafenib	DNMT/HDAC/G‐CSF/DNA and RNA polymerases/TK	DNA methylation/histone deacetylation/Neutrophil growth/DNA synthesis	RR‐AML	Phase I, terminated	NCT02412475	–
Decitabine + vorinostat + vincristine + dexamethasone + mitoxantrone + pegaspargase + methotrexate	DNMT/HDAC/tubulin/glucocorticoid receptor/topoisomerase type II/DHFR	DNA methylation/histone deacetylation/Cell division/DNA synthesis and repair/cell metabolism	ALL	Phase I/Phase II, terminated	NCT01483690	[167]
NTX‐301	DNMT1	DNA methylation	AML MDS	Phase I, not yet recruiting	NCT04167917	–
Decitabine + nivolumab + CDX‐1401	DNMT/PD‐1/DEC‐205	DNA methylation/Immune presentation	AML MDS	Phase I, active (not recruiting)	NCT03358719	–
AZA + nivolumab + ipilimumab	DNMT/PD‐1/CTLA4	DNA methylation/Immune presentation	RR‐AML	Phase II, recruiting	NCT02397720	–
Decitabine + nivolumab + venetoclax	DNMT/PD‐1/BCL‐2	DNA methylation/Immune presentation/apoptosis	AML	Phase I, recruiting	NCT04277442	–
Guadecitabine + atezolizumab	DNMT/PD‐L1	DNA methylation/Immune presentation	AML MDS CMML	Phase I/Phase II, recruiting	NCT02935361	–
AZA + lenalidomide	DNMT/IKAROS	DNA methylation/Immune response	CR‐AML	Phase II, completed	NCT01301820	[168]
AZA + lenalidomide	DNMT/IKAROS	DNA methylation/Immune response	RR‐AML	Phase II, completed	NCT01743859	[169]

### 2.11 DNMT inhibitors

Patients who are not fit to receive standard chemotherapy for AML, or high‐risk MDS patients, may be treated with HMAs [96]. HMAs are generally considered effective and safe [97]. Some have already been approved, and others are currently undergoing clinical trials. The first DNMT inhibitors were discovered in the 1960s [98], and two decades later were introduced into the clinical setting as effective epigenetic modifying agents. In order for these inhibitors not to be cytotoxic, they are usually administered at low, subcytotoxic doses [99]. The two most clinically established first‐generation HMAs that have gained FDA approval are the cytosine analogs decitabine (5‐aza‐20‐deoxycytidine azacytidine, DAC) and azacitidine (5‐azacytidine, AZA). Briefly, they work by being incorporated into the replicating DNA in place of cytosine. The covalent, irreversible bond DAC‐G traps DNMTs, bringing about their degradation by the proteasomal machinery [100]. As this process takes place during the S phase of the cell cycle, DNMTs are not available and aberrant methylation patterns are no longer reproduced in daughter cells, inducing demethylation and reactivation of previously silenced genes, including TSGs [101]. They are the most effective epigenetic therapies to date: Both drugs provide significant clinical benefits, with high overall response rates and improved overall survival in patients with myeloid malignancies [102, 103, 104]. However, it is not clear whether the degree of hypomethylation achieved with drug treatment predicts clinical response in humans [105]. DAC and AZA both reverse DNA methylation patterns and rewire oncogenic transcriptional programs, modulating the expression of the genes that drive neoplastic progression and differentiation, as well as derepressing transcription of TSGs [106], as mentioned earlier. However, treatment with either agent has been reported to cause different gene expression profiles in various leukemia cell lines [105]. Mechanistically, these two drugs act differently. DAC binds to DNA, then, during the S phase of the cell cycle (DNA replication), binds covalently to the DNMT enzymes and inhibits their activity (mainly DNMT1, with higher sensitivity), triggering their further proteasomal degradation. In addition, DAC interferes in the synthesis of new DNA during the S phase, impairing cell proliferation and causing apoptosis [107]. Conversely, AZA preferentially binds the cells' newly synthesized RNA (~ 80–90%, the rest binds to DNA), giving rise to mRNA and protein metabolism disruption and apoptosis. Around 10–20% of AZA is converted to DAC and binds DNA [108]. Nevertheless, and despite their proven efficacy in several hematopoietic malignancies, HMAs are not curative and treatment with them needs to be continuous [101]. Currently, several efforts to bypass this and other limitations and achieve more durable responses and patient remission rates, along with combination therapies, are being investigated by the research community to improve the efficacy of these epigenetic remodeling drugs (Table 2). One such example is given by administering both DNMT inhibitors and histone deacetylase inhibitors (HDACis) [105], given that DNMTs also attract HDACs to the CpG loci they act upon, thus further stabilizing the silencing of the target gene [110]. In Table 2, we summarize some ongoing clinical trials that combine these two therapeutic agents. However, with the development of new and effective HDAC inhibitors arise, like the novel small molecule HDAC6 inhibitor QTX125 for mantle cell lymphoma [111], it will be possible to test new combination therapies in the clinical setting. Another interesting example is that of the FDA‐approved combination of venetoclax (BCL‐2 inhibitor) and DAC or AZA [112] for treating elderly AML patients, which is already the subject of two Phase 3 trials (NCT02993523 and NCT03069352). Finally, second‐generation HMAs are being investigated. One such drug is guadecitabine (Phase 3 clinical trial) [97], which is an HMA that is more resistant to degradation and, consequently, increases both the response rate and treatment efficacy, as reported by Griffiths *et al*. [113].

### 2.12 TET inhibitors

**Fig. 4:**
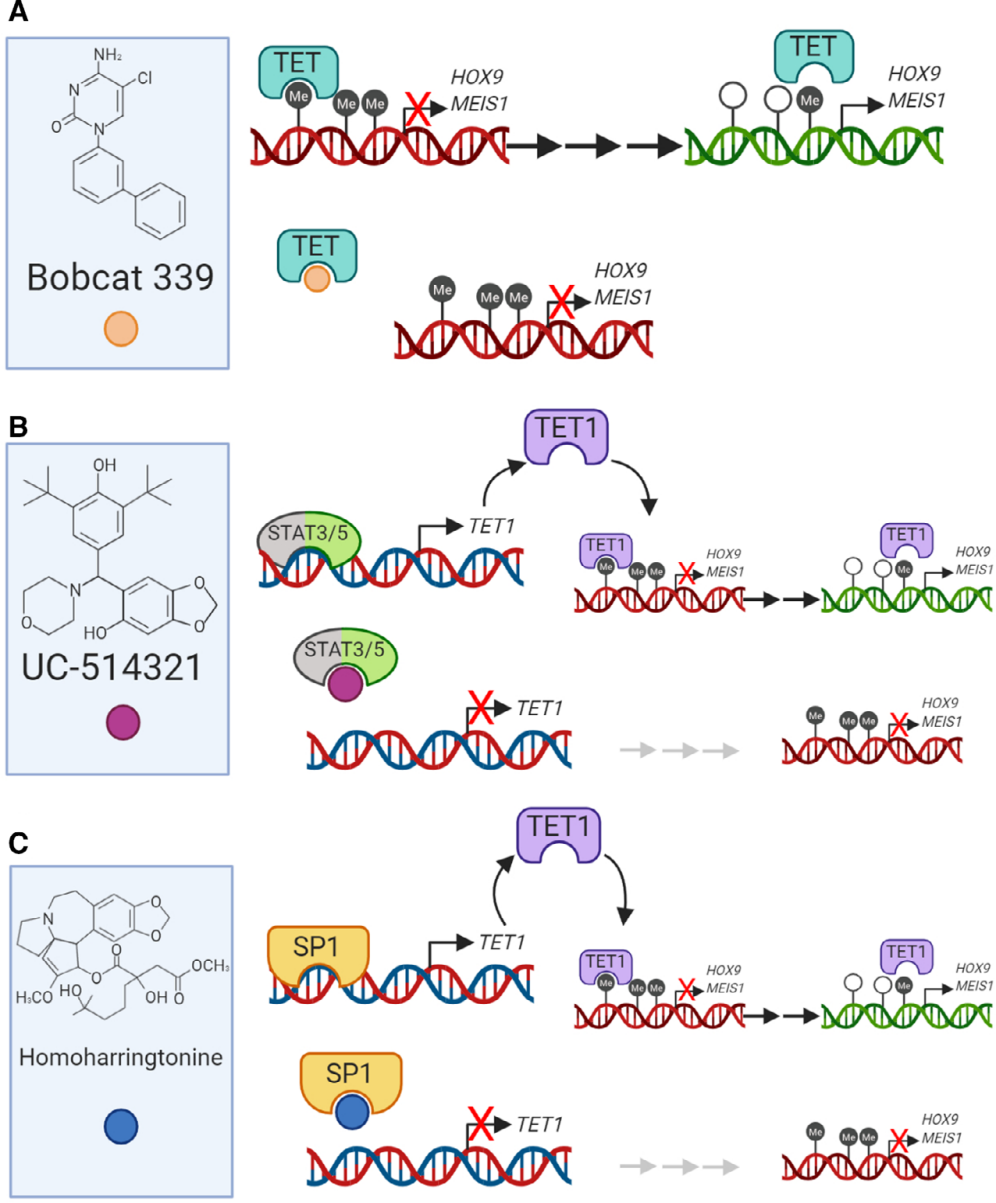
Mechanism of action of TET inhibitors. (A) Direct inhibition. A novel compound discovered by Chua *et al*. [121], Bobcat 339 emerged from a selection of different TET enzyme inhibitors as the most successful on inhibiting the enzyme function by binding its Cl residue on the pocket reserved for Me. The drug blocks both TET1 and TET2 enzymes and does not interact with other methylation enzymes such as DNMTs. (B) Indirect inhibition. JAK/STAT pathway is involved in *TET1* transcription, and STAT inhibitor UC‐514321 seems to stop aberrant TET1 function found in AML according to Jiang *et al*. [122]. (C) Indirect inhibition. SP1 appears to be a transcription factor involved in TET1 transcription, and as in the case of UC‐514321, blocking TET1 transcription and translation in AML gives promising results avoiding the spread of the malignancy.

To our knowledge, no specific TET2 inhibitors have so far had any clinical application. However, TET2 mutations are relatively common in myeloid cancers [42] and are present in 20–25% of MDS, 7–23% of AML, and up to 53% of chronic myelomonocytic leukemia (CMML) patients [114, 115, 116]. TET2 mutations have also been observed in lymphomas (12% of DLBCL patients, predominantly in the GCB subtype) [117]. They occur most frequently in T‐cell lymphomas, specifically, they are present in at least 50% of angioimmunoblastic T‐cell lymphomas (AITLs) [118]. These authors speculated about the possible oncogenic cooperation between TET2 and DNMT3A mutations. The effect of AZA in AITL patients with TET2 mutations was evaluated by Delarue *et al*. [119], who reported an objective response rate to AZA treatment, including complete remission. Further evidence of the clinical utility of TET2 inhibitors was provided by Dominguez *et al*. [120], who observed growth inhibition of TET2‐knockdown DLBCL cells after treatment with a histone deacetylase 3 (HDAC3) inhibitor *in vitro*. This highlights once more, as in the case of HMAs, the clinical utility of combining epigenetic therapies. Even though the study of TET2 inhibitors is currently only at the preclinical stage, several mechanisms of action (MoA) have been proposed. Direct inhibition of TET1 or TET2 was suggested by Chua *et al*. [121], who found that the novel compound Bobcat 339 best inhibited the enzymatic function of TET1/2 by binding to its catalytically active residue. The drug blocked the enzymatic activity of both TET1 and TET2, and did not interact with DNMTs (Fig. 4A). Two other studies demonstrated the indirect inhibition of TET1. Jiang *et al*. [122] showed that the JAK/STAT pathway is involved in TET1 transcription in AML, and by using the STAT inhibitor UC‐514321, they were able to stop aberrant TET1 function *in vitro* (Fig. 4B). Finally, Li *et al*. [123] recently showed that SP1 is a transcription factor involved in TET1 transcription in AML. Using the potent FDA homoharringtonine, they managed to block TET1 transcription and translation while arresting disease progression (Fig. 4C).

### 2.13 DNA methylation signatures as diagnostic and prognostic tools

The reversible nature of epigenetic changes, as opposed to genetic mutations, offers the possibility of using DNA methylation as an attractive therapeutic target. Therefore, several attempts have been made over the past 20 years or so to find aberrant DNA methylation patterns that might have a diagnostic or prognostic value for several hematological cancers. These patterns or signatures may consist of a single gene or a combination of them. The highly dynamic and plastic nature of DNA methylation alterations means that these aberrations very often change over time, that is, over the course of a disease [124] and with age [125]. In this way, some of these patterns are positively selected by cancer cells to favor neoplastic development, as is certainly the case for both MDS and AML [125]. Thus, the signatures found so far are usually stage‐ or age‐specific, as well as often subtype‐specific (due to the great heterogeneity of methylation patterns in these malignancies). As a good example of a DNA methylation signature, Aggerholm *et al*. [124] found four genes whose promoters were often hypermethylated in MDS (*p15^INK4B^*, *HIC1, CDH1*, and *ERα*). While hypermethylation of three or more of these genes occurred more frequently in advanced MDS, it became clear that promoter hypermethylation of one or more of these four genes was predictive of poor prognosis in patients with early‐stage MDS. Furthermore, *p15^INK4B^* hypermethylation in patients with early MDS was significantly associated with transformation to AML. Notably, there was no significant variation in the promoter methylation status over time (a period of 1132 days, with a median 284 days between measurements) [124]. Patient age was not significantly associated with these observations either. Considered as a whole, this and the other signatures described in the literature suggest that they may have profound clinical implications, as the respective disease subtype or patient group could be treated with the HMAs described above (or in combination with other epigenetic drugs, Table 2). Nevertheless, it should be pointed out that even though the response to epigenetic drugs might be monitored, for instance, by measuring signature reversal throughout the course of treatment, there are currently no cost‐effective tools available in the clinic for that purpose. Some authors have already proposed that pretreatment methylation levels of long interspersed element (LINE‐1) in elderly AML patients treated with AZA are sufficient to predict the clinical response. In this study, lower baseline levels of LINE‐1 methylation were noted in those patients who went on to achieve a complete or partial remission [126]. However, the low patient number was a key limiting factor of this study,larger cohorts need to be studied in order to validate this relationship. In fact, the reliable assessment of HMA response in the clinical setting remains a topic of intense debate [105, 127].

### 2.14 DNA methylation and the (chemo)immunotherapy response

Resistance to HMAs is frequent in MDS [128] and other hematological malignancies, as is, to a lesser extent, nonresponsiveness [129, 130]. Treatment of MDS or AML patients with HMAs may induce immune reaction alterations in those patients [131], indicative of a putative role of this immune deregulation in HMA resistance. For instance, it has been shown that treatment of DLBCL cells *in vitro* with low‐dose DNMTi sensitized them to standard chemotherapy, mainly through promoter demethylation of SMAD1 [132]. Consequently, a Phase I clinical study was performed combining AZA with chemoimmunotherapy, in which demethylation of SMAD1 could be tracked and confirmed pre‐ and post‐treatment, reaffirming the initial study's identification of this gene as a candidate chemosensitization agent [132]. Despite this and other research efforts, the precise molecular mechanisms linking resistance/refractory behavior and the immune landscape reshaping of these neoplastic cells are not well understood. A pioneer study by Yang *et al*. [133] showed how MDS and AML patient samples and leukemia cell lines treated with demethylating agents caused upregulation (increased expression) of the immunosuppressive ligands PD‐L1, PD‐L2, and their (immunosuppressive) checkpoint receptor PD‐1, including *in vitro* PD‐1 demethylation and subsequent reactivation. The authors proposed that this indicated a possible mechanism for resistance to HMAs. They also noted that MDS/AML bone marrow blasts were positive for PD‐L1, whereas the stroma/nonblast cellular compartment (i.e., the TME) was positive for PD‐1, leading them to speculate about the sensitivity of these cell lines to ICIs. Analogously, Srivastava *et al*. [134] showed that HMA therapies can reactivate testicular cancer antigens and induce robust immune recognition by T cells in AML patients. Overall, these and other results pointed toward the possible use of ICIs to improve outcomes in leukemia and lymphoma, either alone or in combination with HMAs, since HMAs have an affinity for immunosensitive neoplastic cells, and therefore confer on them a greater sensitivity to ICIs. This link between immune regulation and HMAs has been explored by several other authors [135, 136] and is the subject of current research efforts [137]. For instance, it has been recently reported that apart from modulating the tumor's immune responses, HMAs might act directly on exhausted T cells to reverse the onset of exhaustion and restore their cytotoxicity activity [138]. At the time of writing, several clinical trials involving both HMAs and ICIs are already in progress (Table 2). Another promising combination therapy is the joint use of HMAs and immunomodulatory drugs (IMiDs). The latter are a set of drugs that have initially been highly success in the treatment of MM. They have antiproliferative effects and costimulate T and NK cells, enhancing anti‐MM immune activity *in vitro* [139]. While their *in vivo* effects are not yet clear, two potent IMiDs, lenalidomide and pomalidomide, are currently under active investigation, for the treatment not only of MM, but also of other hematological neoplasms.

## 3 Conclusions and perspectives

DNA methylation is a highly dynamic process that aids normal hematopoiesis control. The rapid progress in high‐throughput sequencing technologies (WES, WGS, EPIC methylation arrays, and WGBS) over the last decade has enabled researchers to identify many genetic and epigenetic aberrations in different types of blood cancers. These diseases also usually present a low (or very low, in the case of AML, for example) somatic mutation rate in their genomes, and are often considered to be ‘epigenetic malignancies’. When DNA methylation anomalies arise during hematopoiesis, they often trigger the initiation and progression of hematological cancers. However, DNA methylation aberrations do not function exclusively, often acting in synergy with other epigenetic lesions, showing that the epigenetic regulation of cell development and fate is a very complex and intricate process. In this review, we have summarized the current knowledge about the main DNA methylation remodelers (DNMTs, TETs, and IDHs), their relationship with other epigenetic effectors, and the possible treatments and clinical applications. HMAs have emerged as promising agents to treat several hematological neoplasms, due to their capacity to reverse aberrant methylation patterns. However, because HMAs inhibit DNMTs, they are highly cytotoxic at high doses, causing an indiscriminate global loss of methylation. Indeed, inclusion of the HMA agents AZA and DAC in the RNA or DNA at high doses produces severe DNA damage and inhibits protein synthesis, making these compounds highly toxic to patients. Therefore, optimal, subcytotoxic doses must be applied. Survival data [103, 104] currently suggest that AZA may be clinically superior to DAC, but the lack of systemic clinical trials comparing the two regimes makes this comparison difficult and the results unclear [140]. However, the observed higher efficacy of AZA might be due precisely to its ability to incorporate into the RNA [141]. The development of specific catalytic inhibitors of individual DNMT enzymes, or targeting specific DNMT‐containing complexes, is of fundamental importance to improving the clinical efficacy of current HMAs [142]. We also mentioned the emerging evidence supporting the use of combinations of epigenetic drugs, and noted that these combinations may be important in the treatment of hematological malignancies (Table 2). As mentioned above, clinical trials combining HMAs and ICIs are already underway (Table 2). Their merit is supported by the fact that DNA methylation abnormalities may actually have profound effects on the cytotoxicity of certain immune system cellular subtypes (e.g., by inducing the T‐exhausted phenotype). While HMAs and HDACis alone may have proimmunogenic effects, combinations of them have also been shown to increase the infiltration and activation of effector immune cells while reducing the infiltration of immunosuppressive cells [143]. Given the increasing evidence linking HMA action and DNA damage, and the frequent co‐occurrence of DNMT mutations and other genetic lesions, we speculate that synthetically lethal approaches might be an option for treating certain types of blood cancers. Recent studies have demonstrated a synergistic interaction between HMAs and PARP inhibitors [144, 145]. Valdez *et al*. [146] have recently shown that the combined use of HMA, HDACi, and PARPi resulted in extensive DNA damage, double‐strand breaks (DSBs), and apoptosis. Furthermore, evidence is accumulating that links the microsatellite instability (MSI) phenotype of hematological tumors to the disruption of the mismatch repair pathway by promoter hypermethylation of the pathway's own genes [147]. Thus, it is reasonable to speculate further that, as observed in solid tumors, the MSI phenotype might confer some hematological neoplasms with higher rates of ICI responses, through enhancement of neoantigen presentation [148]. In conclusion, other epigenetic regulators, such as miRNAs or lncRNAs, are also attractive putative therapeutic targets, since they control several pathways that are dysregulated in hematological malignancies and are often linked to DNA methylation in these diseases. Further research in this area is likely to yield highly valuable results.

## Conflict of interest

ME is a consultant for Ferrer International and Quimatryx. The other authors declare that they have no conflict of interest.

## Author contributions

All authors contributed to writing the manuscript, read, edited, and approved the final manuscript.
